# Improvement of Early Strength of Cement Mortar Containing Granulated Blast Furnace Slag Using Industrial Byproducts

**DOI:** 10.3390/ma10091050

**Published:** 2017-09-07

**Authors:** Jin-Hyoung Kim, Han-Seung Lee

**Affiliations:** 1 Department of Architectural Engineering, Hanyang University, 68 Munam 1-ga, Dong Nam-gu, Cheonan-si 31065, Chungcheongnam-do, Korea; bestkjh007@naver.com; 2 Department of Architectural Engineering, Hanyang University, 55, Hanyangdaehak-ro, Sangrok-gu, Ansan-si 15588, Gyeonggi-do, Korea

**Keywords:** early strength accelerator (ESA), granulated blast furnace slag, early strength development, industrial by-product

## Abstract

In the field of construction, securing the early strength of concrete (on the first and third days of aging) has been an important problem in deciding the mold release time (i.e., shortening the construction time period). Therefore, the problem of reduced compressive strength in the early aging stage caused by mixing granulated blast furnace slag (GBFS) with concrete must certainly be resolved. In this study, we conduct experiments to explore methods for generating a concrete that develops an early strength equivalent to that of 100% OPC. The objective of this study is the development of an early-strength accelerator (ESA) made from an industrial by-product, for a GBFS-mixed cement mortar. This study also analyzes the mechanism of the early-strength generation in the concrete to evaluate the influence of the burning temperature of ESA on the optimal compressive strength of the concrete. According to the results of the experiment, GBFS, whose ESA is burnt at 800 °C, shows an activation factor of 102.6–104.7% in comparison with 100% OPC on the first and third days during early aging, thereby meeting the target compressive strength. The results of the micro-analytic experiment are as follows: ESA showed a pH of strongly alkaline. In addition, it was found that the content of SO_3_ was high in the chemical components, thus activating the hydration reaction of GBFS in the early age. This initial hydration reaction was thought to be due to the increase in the filling effect of the hydrate and the generation of C-S-H of the early age by the mass production of Ettringite.

## 1. Introduction

Currently, eco-friendly and low-carbon products are being actively developed and efforts are being made to innovate their manufacturing processes in all the industrial fields in accordance with the green-growth policies implemented worldwide. In the construction industry, cement is an essential binder used for manufacturing concrete. However, the volume of limestone mining is expected to reach its limit soon, so that a rise in the manufacturing cost of cement is inevitable in the future because of the regulations on greenhouse gas emissions. Notably, the construction industry emits approximately 7–9% of CO_2_ globally, and therefore, more efforts have been made by the industry to reduce carbon emissions [1,2,3,4]. To establish an eco-friendly structure of the construction industry, priority should be given to its environmental load. It is crucial to more vigorously apply environmental load standards to the construction industry in terms of reducing the energy use, using low-carbon construction materials, and recycling industrial by-products and wastes [5,6,7]. In particular, Granulated Blast Furnace Slag (GBFS), a by-product of the steel industry, has a relatively stable chemical composition and useful latent hydraulic property. GBFS also has a significantly lower per-unit CO_2_ emission in comparison with Ordinary Portland Cement (OPC), and it offers diverse technological, economical, and environmental advantages. Therefore, it is very important to use it more actively as an admixture for concrete. In view of the above, the use of GBFS is expected to expand more widely worldwide [8,9,10,11,12,13].

Portland Blast Furnace Slag Cement (PBFSC), which is a mixture of OPC and 20–40% of GBFS, is the most typical type of GBFS used in the construction industry. More recently, High-volume Blast Furnace Slag Cement (HBFSC), whose GBFS content is heightened to 60–70% by applying a cutting-edge technology, is being used for the reduction of the heat of hydration and improvement of seawater and chemical resistance performances [6]. However, GBFS-mixed concrete also has certain disadvantages such as a delayed setting and substantial decrease in the compressive strength in the early aging stage in comparison with 100% OPC. That is why the range over which GBFS-mixed concrete can be used is limited despite its several advantages [10,11,12,13,14]. In the construction field, securing concrete with an early strength (on the first and third day of aging) is a particularly crucial factor that determines the time of removal of the forms (or the shortening of the construction time period). Consequently, reduction in the early strength of concrete caused by the mixing of GBFS must certainly be resolved.

The methods for improving the early strength of a GBFS-mixed concrete include increasing the fineness of the powder (8000–10,000 cm^2^/g) and using an early-strength admixture [15,16]. However, these methods are not economically feasible because of the drying shrinkage and rise in costs, and so, their applications are limited to special concrete only. Recently, studies have been conducted internationally regarding approaches for improving the early strength of concrete such as the use of alkali-activated blast furnace slag cement and Geopolymers [1,3,4,17,18,19]. However, these methods are limited in their practical applications in the construction industry. In addition, a majority of these studies were focused on the strength of concrete on the third and seventh day of aging, as well the as long-age-st**r**ength, rather than the early-strength (on the first and third day of aging). They also failed to clearly identify the mechanism of the hydration reaction triggered by alkali-activation.

The objective of this study is to form an economical Early-Strength Accelerator (ESA), which can be mass-produced by applying a source technology, for a material that helps in the development of its early-strength (on the first and third day of aging) earlier than 100% OPC. The experiment for this study was conducted on the basis of the results obtained from preceding studies [20,21].

The industrial byproducts used in this study are: tap water, which is refined, and the remaining sludge. A High content of SiO_2_-Al_2_O_3_ (Sludge), Chemical Gypsum (CG) is generated during the manufacturing process of titanium oxide [22,23]. Limestone Powder (LP) [24] that affects the hydration reaction during early aging was selected for the experiments in this study. An ESA with different chemical substances and different ratios of the admixture was placed in a pilot calcination furnace and burnt at different temperatures (700–1100 °C) in the form of a clinker. The burnt ESA was mixed with OPC and GBFS to experimentally investigate the changes in properties and development of compressive strength at different points of aging. Then, the cement paste experiment was performed to analyze and compare the influence of the hydration reaction of GBFS [25] and the chemical properties. The results of this study could be utilized as the basic reference for the material technology for generating concrete that uses the ESA developed herein. In addition, the environmental impact evaluation of ESA energy consumption and the emission calculation utilized in this study have been carried out as in previous research [26].

## 2. Experimental

### 2.1. Experimental Plan and Mortar Mix

After individually burning (0–1100 °C) each of the raw materials selected in this study, their chemical compositions and substances were analyzed. The experiment was conducted separately for the mortar level and cement paste level. The mortar experiment was designed to identify the flow performance and property of compressive strength development.

**Figure 1:**
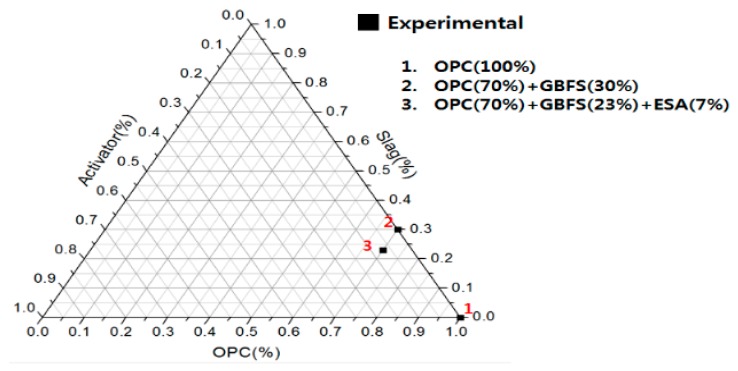
Mixing ratio of powders for different materials (%).

The experiment with the cement paste was designed to identify the mechanism of the hydration reaction on the cement paste resulting from the use of ESA and mechanism of early compressive strength development via microanalysis. Mixtures of the substances used in the experiments are displayed in Figure 1.

### 2.2. Materials for Experiment (Raw Materials)

The type of OPC used possessed a fineness of 3450 cm^2^/g. For PBFSC, three types of GBFSs were mixed at 30% of the content (including 3.7% of natural anhydrous gypsum).

The basicity (b = (CaO + MgO + Al_2_O_3_)/SiO_2_) was 1.79 and fineness was 4330 cm^2^/g. In consideration of the economic feasibility, OGE (70% OPC + 23% GBFS + 7% ESA) without natural anhydrous gypsum was used for the experiment. Its chemical composition is demonstrated in Table 1.

CaSO_4_ + 2H_2_O (1)

Based on Table 2, a sludge with a high content of SiO_2_–Al_2_O_3_, used as a raw material for the development of an ESA, includes 60–70% moisture. It also contains chemical components such as SiO_2_ (25–46%), Al_2_O_3_ (25–38%), and Fe_2_O_3_ (2–5%). When organic impurities are removed by burning, the sludge shows an advantage in contributing to an improved early-strength development of concrete. CG is a by-product generated during a chemical reaction that neutralizes acidic sulfur to lime, and whose main components are CaO (32–39%) and SO_3_ (45–50%). When the ignition is lost and impurities are removed, LP is the main component of OPC, which exists as CaCO_3_. Usually, it is converted into CaO during decarboxylation at a temperature > 900 °C. The pH of LP increases from 6 before burning to 12 after burning. It plays the role of a catalyst for the early hydration of Ca(OH)_2_ crystals and it accelerates the hydration of OPC particles [24]. It was selected as one of the raw materials for this study as it can help draw the optimal mix of raw materials for different burning temperatures, because it is a strong alkaline substance that affects the hydration process of GBFS [25]. Table 3 displays the chemical composition of the ESA that was burnt (700–1100 °C) with a 40:30:30 ratio of CG, sludge, and LP in the pilot kiln. Due to the mixing of the LP, the pH of the ESA is strongly alkaline, which promotes the initial hydration of PBFSC. Table 4 compares the chemical composition of 7% ESA with PBFSC compared to OPC (70% OPC + 23% GBFS + 7% ESA: OGE). Also, each production time was 90 min from the injection time through the firing line. Changes in the pH of ESA resulted from the firing temperature of LP in ESA.

### 2.3. Method of Experiment and Items for Measurement

Table 5 shows the mix, compressive strength, and flow of mortar and Table 6 displays the mix of cement paste.

In the mixtures used for the experiments (70 wt % OPC, 23% GBFS 23% mass, 7% ESA mass), the ratio of each binder and fine aggregates was set at 1:3. The ratio of water to binder was fixed at 50%. The 7% ESA mixture against the mass of cement was experimented at five levels (700–1100 °C). Curing was conducted in an isothermal-isohumidity room at a curing temperature of 20 °C ± 2 °C and with a high relative humidity of 95%. After one day of aging, the cured water was removed. The compressive strength was tested on the 1st, 3rd, 7th, 14th, and 28th day of aging and the compressive strength was determined by calculating the average of three compressive strength values measured for the day. Furthermore, its basic property was compared using the measuring flow. The cement paste experiment was conducted at seven levels: OPC, PBFSC, and OGE1 to OGE5. The ratio of water to cement (W/C) was fixed at 50%. The method of curing was the same as that of the mortar experiment. Table 7 displays the raw materials for ESA and the items measured in the mortar experiment and cement paste experiment.

## 3. Result of Experiment and Discussion

### 3.1. Analysis of Crystal Structure for Different Burning Temperature of Raw Material (ESA) (XRD)

**Figure 2:**
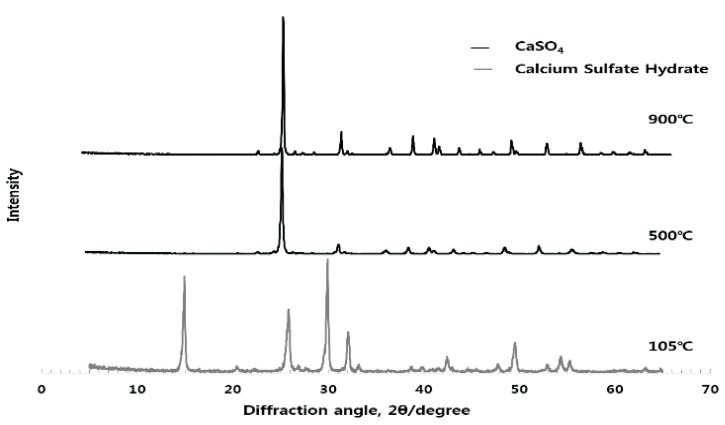
Quantitative analysis of the crystal structure of CG at different burning temperatures.

**Figure 3:**
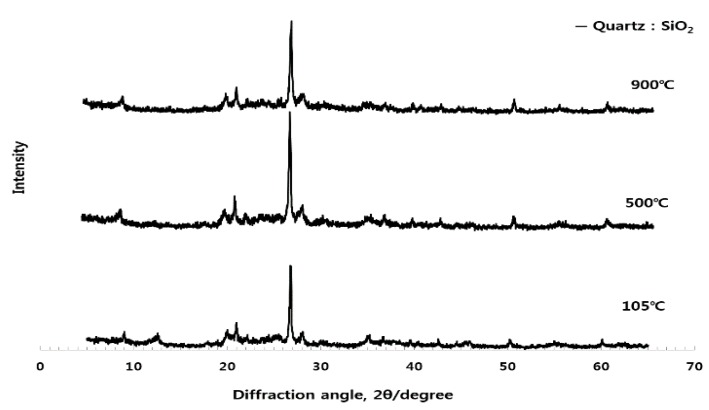
Quantitative analysis of the crystal structure of sludge at different burning temperatures.

**Figure 4:**
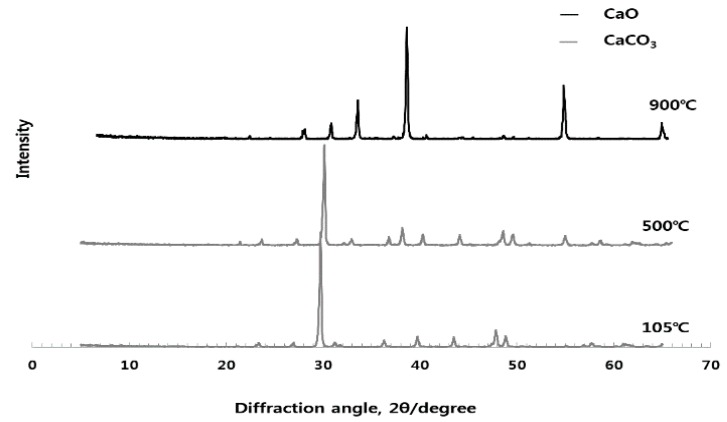
Quantitative analysis of the crystal structure of LP at different burning temperatures.

As can be seen from Figure 2, Figure 3 and Figure 4, the experiments in this study are conducted to analyze the influence of each raw material on the activation of ESA. When the CG, sludge, and LP are burnt individually, CG is dehydrated at 105 °C, as demonstrated by the appearance of the peak of gypsum hemihydrate. Then, at the burning temperature of 500 °C or higher, it is turned into anhydrous gypsum with a high solubility. The use of CG appears to enable early-strength development by inducing the generation of C-S-H and C-A-H of GBFS. The peak of SiO_2_ was high in sludge. In the chemical composition of the sludge, the content of the SiO_2_ component was 45–55. LP existed as CaCO_3_ at 800 °C, and was then decarbonated at 900 °C and turned into CaO, which is a strong alkaline substance that accelerates the generation of Ca(OH)_2_ when reacting with water. It is thought to enable early hydration by removing the oxide coating of GBFS during the early stage.

### 3.2. Mortar Experiment

#### 3.2.1. Flow of Mortar

**Figure 5:**
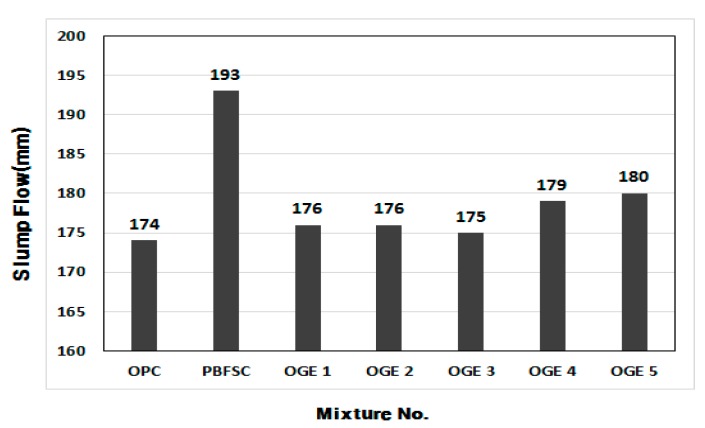
Measured value of flow of OGE (mm) at different ESA burning temperatures.

Figure 5 displays the measured values of the mortar flow at different burning temperatures of ESA. The value of flow for 100% OPC mortar was 174 mm, the lowest among all the experiment factors. The one with the largest flow was PBFSC, which showed a value more than 11% higher than the flow of 100% OPC. The flow of ESA-mixed OGE factors was 0.5–1.0% larger than the flow of OPC, which is almost the same as the flow with OPC. Further study on the constructability of concrete in its fresh state, including time-lapsed changes in the value of flow, will have to be conducted in the future.

#### 3.2.2. Compressive Strength (MPa) and Activity Factor (%) of OGE at Different ESA Burning Temperatures

**Figure 6:**
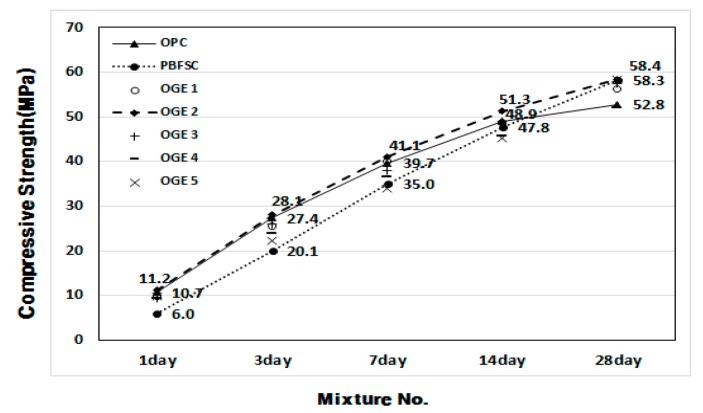
Compressive strength of OGE (MPa) at different aging times and different ESA burning temperatures.

**Figure 7:**
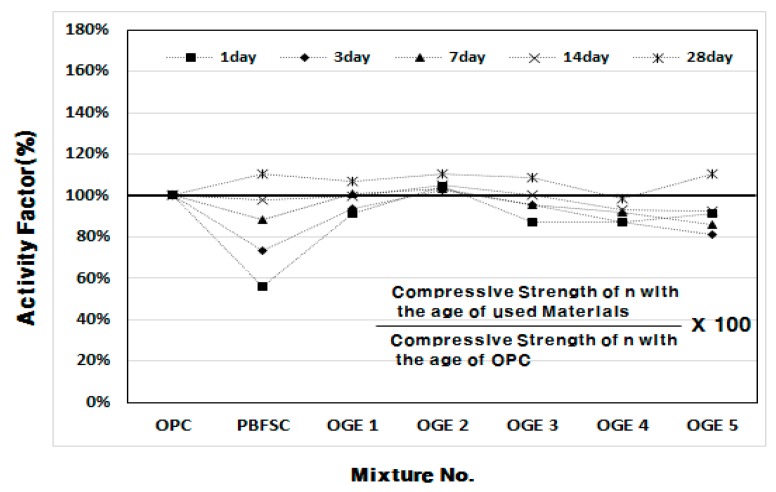
Activity factor of OGE (%) at different aging times and different ESA burning temperatures.

Figure 6 and Figure 7 show the plots of the compressive strength development of OGE mortar at different ESA burning temperatures. The three compressive strengths for each age are shown as average values. When the compressive strength is measured, the activity factor of PBFSC is 56.1–73.4% of the factor for 100% OPC from the first to third day of early aging. Such low early-strength poses a serious problem in the construction industry because it restricts the shortening of the construction period. For OGE with the ESA, a higher burning temperature did not result in a higher compressive strength, but caused a strength decrease. However, OGE 2 that employed OGE at 800 °C, burns at a low temperature and shows an activity factor of 102.6–104.7% that fulfills the criteria for strength during the early aging stage (on the first and third day of aging). Interestingly, the compressive strength at a long-term age on the 28th day is also satisfactory. Therefore, the use of OGE at 800 °C is thought to not only activate the reaction of GBFS earlier, but also affect the compressive strength during long-term aging. Such a result also indicates that burning at a low temperature reduces the requirement of energy use.

### 3.3. Cement Paste Experiment

#### 3.3.1. Setting Time of Cement Paste

**Figure 8:**
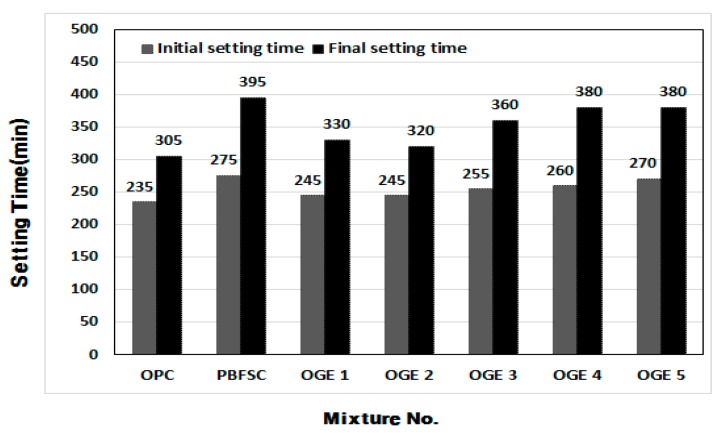
Setting time of OGE at different ESA burning temperatures.

Figure 8 shows the setting time of OGE cement paste at different ESA burning temperatures. The time of initial setting and final setting was the earliest for 100% OPC, respectively. The initial and final settings of PBFSC were 40 and 90 min later than those of 100% OPC. These settings indicate that the reaction of cement is substantially decelerated, resulting in a delay in the setting time. The speed of the reaction of OGE is slowed following an increase in the ESA burning temperature. For OGE2 which showed a satisfactory compressive strength, the same cement reaction was observed. The Initial setting time was 245 min and the Final setting time was 320 min.

#### 3.3.2. Change in Chemical Composition of ESA and OGE according to Burning Temperature

**Figure 9:**
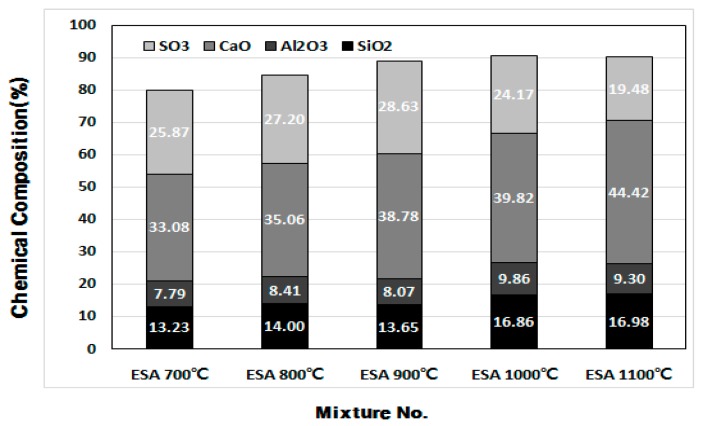
Change in ESA chemical composition (%) at different burning temperatures.

**Figure 10:**
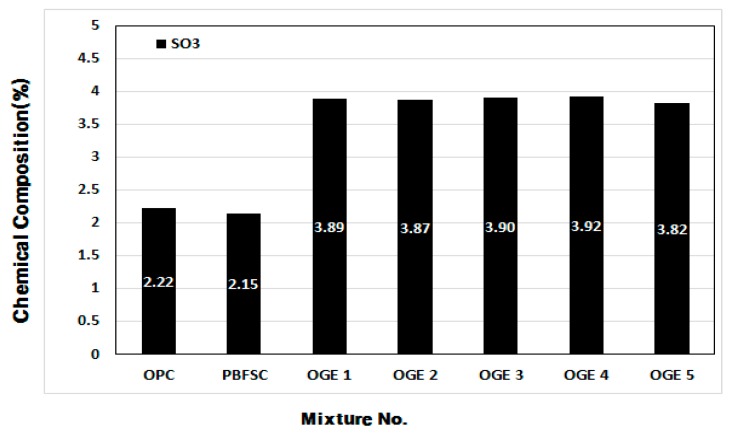
Scope of chemical composition (%) of SO_3_ at different ESA burning temperatures.

Figure 9 and Figure 10 demonstrate the changes in the chemical composition as a function of the ESA burning temperature and contents of the chemical composition of ESA-mixed OGE. The major components that have a valid effect on early aging are compared in the figures.

Among the major chemical components of ESA, the SO_3_ content decreases, while the CaO and SiO_2_ content increases as the burning temperature increases. Such changes are thought to be attributed to the change in the chemical composition following the decarboxylation of LP and combustion of CO_2_. In comparison with OPC, the SO_3_ content in PBFSC is higher by approximately 2%. The SO_3_ content in OGE with ESA burnt at different temperatures is also greater by approximately 2% when compared to PBFSC.

Furthermore, OGE has a similar chemical composition regardless of the burning temperature of ESA, which is considered to be due to the insignificant share of ESA in the entire OGE, at about 7%.

#### 3.3.3. Analysis of Crystal Structure (XRD) according to Use of ESA

**Figure 11:**
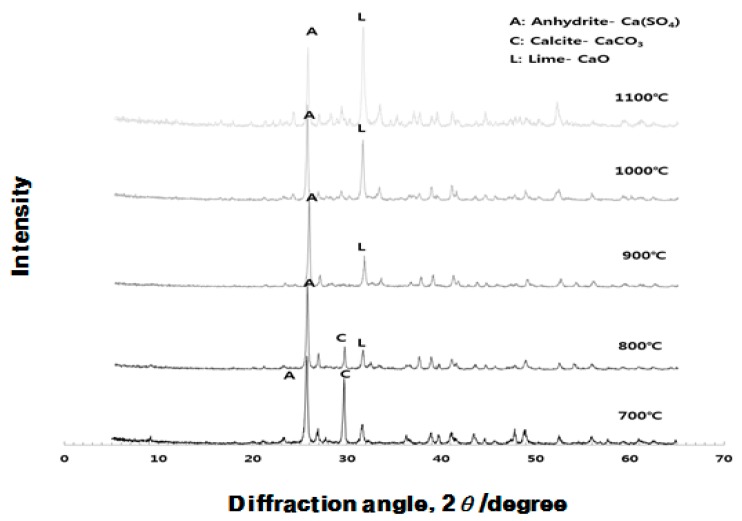
Quantitative analysis of the crystal structure of ESA at different burning temperatures.

**Figure 12:**
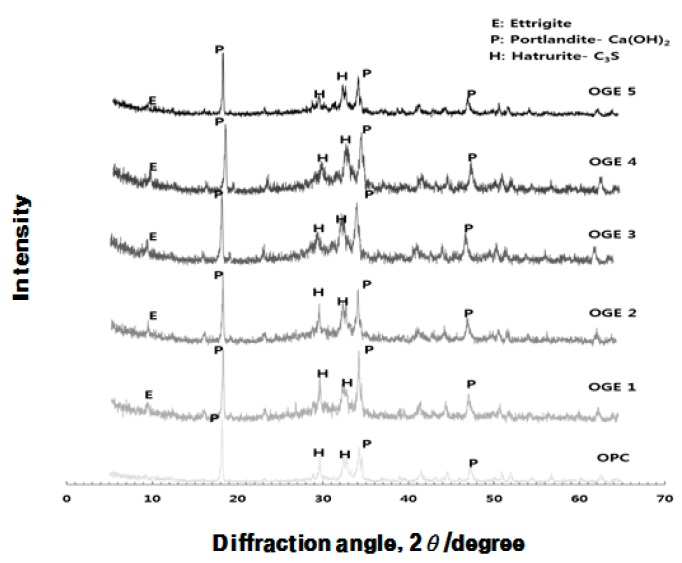
Quantitative analysis of the crystal structure of OGE with aging time at different ESA burning temperatures (1st day).

**Figure 13:**
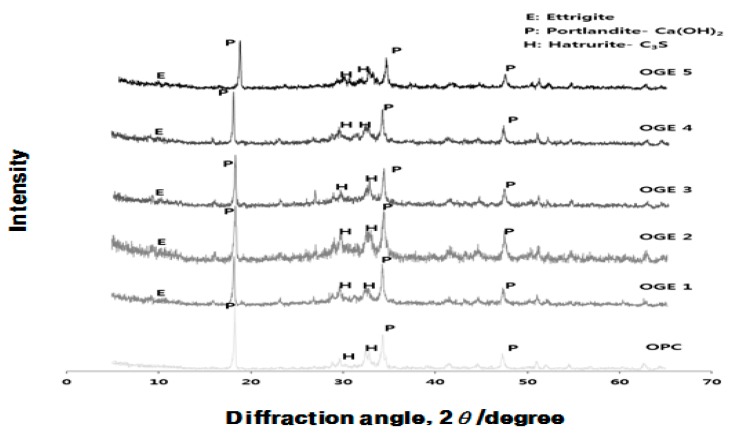
Quantitative analysis on the crystal structure of OGE by aging time at different ESA burning temperatures (3rd day).

Figure 11, Figure 12 and Figure 13 display the results of the analysis of the XRD crystal structure, which was obtained in order to identify the influence that OPC and ESA-mixed OGE paste have on the hydration chemical characteristics.

Figure 11 shows that as the crystal structure changes as a function of the burning temperature of ESA, the peak is generally higher for the anhydrous gypsum and it turns into the peak of CaCO_3_ at 700–800 °C and then into the peak of CaO at 900 °C or higher. ESA-mixed OGE shows the best compressive strength at 800 °C in terms of the burning temperature. In this region, XRDs of anhydrous gypsum, CaCO_3_, co-exist.

When the burning temperature increases, the main peak point is turned into CaO from the anhydrous gypsum. The higher the CaSO_4_ content compared with the CaO content, the more effective that CaSO_4_ is as an effective activator for improved compressive strength in the early aging stage.

According to Figure 12 and Figure 13, hydration of the hardened ESA-mixed OGE cement paste is suspended after the first and third days of early aging and the hydrates are measured instead. OPC has the highest Ca(OH)_2_ peak, showing a typical hydration product of OPC. In contrast, ESA-mixed OGE generates Ettringite as a hydration product. The generation of Ettringite and C-S-H in early aging is thought to be the cause of the compressive strength development from its filling effect. On the third day of aging, C_3_S, which is a non-hydration product, decreases and Ca(OH)_2_ increases. The peak of Ettringite is generally reduced on the third day of aging, which is because when gypsum runs out in the process of hydrate generation of cement, it comes to exist as a monosulfate, which is a stable hydrate.

#### 3.3.4. Stability Test (Free-lime Quantitative Analysis)

**Figure 14:**
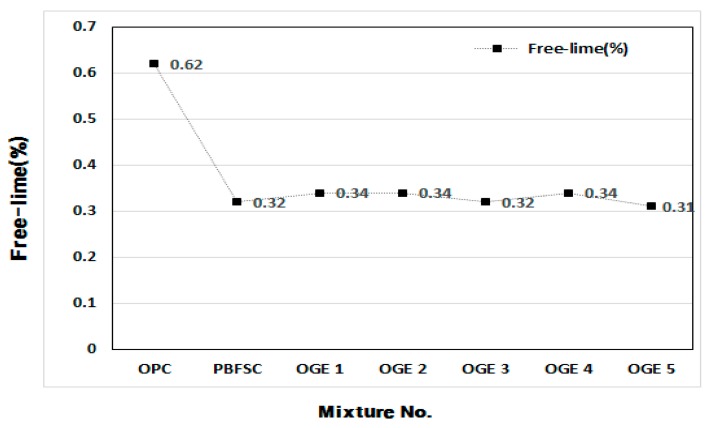
Measured value of free-lime in OGE at different ESA burning temperatures.

A test (free-lime) on the stability of OGE at different burning temperatures of ESA was conducted. It was based on the formula below, by using the Ethylene Glycol method. As displayed in Table 8 and Figure 14, the expandability of OGE and size of the degree of cracks compared with those of OPC. The largest amount of free-lime existed in OPC and the amount was 50 to 54.8% in ESA-mixed OGE, in comparison with OPC. Such a result indicates that OGE is stable for expandability and cracks are caused by free-lime. Generally, the content of free-lime in OGE is similar for different burning temperatures of ESA.

(2) $$\mathrm{Free}\;\mathrm{lime}\left(\%\right)=\frac{V\text{×}F\text{×}K}{Sample\;weight\left(g\right)}\times 100$$

(3) V: 0.1 N HCl Consumption (mL)F: 0.1 N HCl FactorK: 0.1 N HCl 1 mLAn amount corresponding to CaO(1 mL=0.002804 g CaO)

#### 3.3.5. Result of SEM Measurement of OGE for Different ESA Burning Temperatures

**Figure 15:**
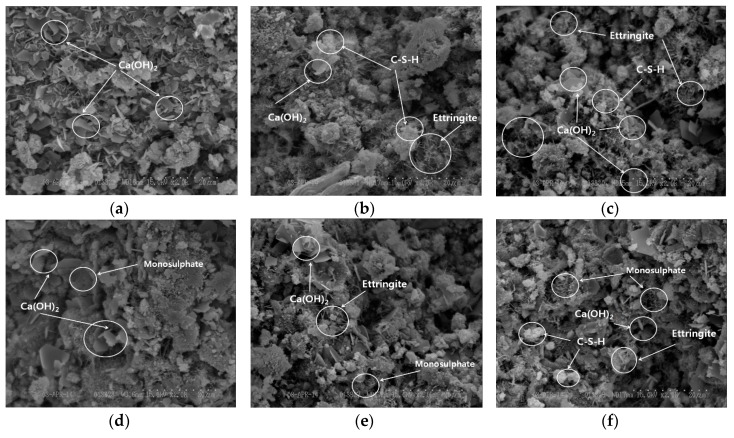
Result of the measurement of OGE as a function of aging time at different ESA burning temperatures (×2000 magnification). (**a**) OPC (one day); (**b**) OGE 1 (one day); (**c**) OGE 2 (one day); (**d**) OPC (three days); (**e**) OGE 1 (three days); (**f**) OGE 2 (three days).

Figure 15 displays the SEM pictures (×2000 magnification) of OPC, ESA-mixed OGE 1, and OGE 2 on the first and third day of aging. For 100% OPC, a large amount of Ca(OH)_2_ crystals are generated after the reaction of C_3_S with water, which is correlated with the peak measured in the XRD analysis. In OGE 1 and OGE 2, the generation of Ca(OH)_2_ and C-S-H was observed. These are generated from the chemical bonding involving Ca^2+^ around the C_3_S particle. In OPC, a monosulfate was observed on the third day of aging. Generally, after one day of aging, gypsum is exhausted and turns into a low sulphate. The SO_3_ content in OGE 1 and OGE 2 is higher than in OPC, as shown in the table of chemical analysis, and SO_3_ is still observed on the third day of aging. In addition, their compressive strength is similar to that of OPC. OPC is thought to be attributable to the effect of GBFS, which has a higher level of fineness than OPC, as well as to the speed of hydration accelerated by the ESA that has a strongly alkaline pH. Another reason for this may be that the active C-S-H hydration reaction has a greater impact on Ettringite and the compressive strength than the generation of a large volume of Ca(OH)_2_ when compared with 100% OPC on the first day of aging.

## 4. Conclusions

- In the mortar experiment, the compressive strength decreased as the sintering temperature of ESA increased. The use of OGE 2 showed an equivalent compressive strength in OPC and early age. In addition, the compressive strength at 28 days was also excellent.
- When the setting time was measured in the cement paste experiment, the OPC reaction was the fastest. As the burning temperature rises, the content of SO_3_ decreases, leading to a rise in the content of CaO and SiO_2_.
- In the XRD and XRF analysis, an increase in the early compressive strength development of OGE was caused by the continuous generation of Ca(OH)_2_ from the pH of the strongly alkaline ESA. In addition, the early strength development was also attributable to the generation and filling effect of C-S-H and Ettringite from the supply of SO_3_.
- In the stability test, ESA-mixed GBFS was found to be stable in terms of excessive expansion and cracks were generated because of the existence of free-lime when applying a construction technology such as steam curing. As the content of free-lime in it is lower than that in OPC, ESA-mixed GBFS is thought to be available when applying the steam curing method such as Precast and when manufacturing special products.
- In conclusion, ESA developed by using an industrial by-product and industrial waste could be used to supply SO_3_ and apply alkaline-activation to improve the compressive strength at the mortar level, as a way of improving the early strength of GBFS-mixed concrete, which usually has the problem of a low early-strength.

## Figures and Tables

**Table 1:** Chemical compositions of cement and GBFS (%).

Raw Materials	Blaine (cm^2^/g)	Ig. Loss	SiO_2_	Al_2_O_3_	Fe_2_O_3_	CaO	MgO	Na_2_O	K_2_O	SO_3_
OPC	3450	1.54	21.58	4.77	3.43	62.59	2.59	0.11	0.85	2.22
GBFS1 ^1^	4330	0.13	34.56	15.30	0.33	42.71	3.90	0.19	0.36	1.98
GBFS2 ^2^	4325	0.12	36.1	15.24	0.45	43.33	4.16	0.21	0.49	0.01

^1^ Natural anhydrous gypsum mixed 3.7%; ^2^ Natural anhydrous gypsum mixed 0.0%.

**Table 2:** Change in chemical composition of individually burnt raw materials according to the type of activator (%).

Materials ^1^		Ig. Loss	SiO_2_	Al_2_O_3_	Fe_2_O_3_	CaO	MgO	Na_2_O	K_2_O	SO_3_	pH
Sludge	105 °C	19.42	43.16	23.92	6.07	0.85	1.60	0.58	2.27	0.55	7.6
	600 °C	4.25	50.72	29.71	6.76	0.96	1.96	0.80	2.61	0.51	7.7
	900 °C	1.29	52.35	31.22	6.72	0.94	2.02	0.76	2.65	0.33	7.7
	1100 °C	0.00	53.38	30.73	7.20	1.03	2.06	1.00	2.76	0.10	7.7
CG	105 °C	11.66	1.89	0.49	0.84	31.72	1.33	0.11	0.38	47.84	7.1
	600 °C	4.14	2.20	0.60	0.92	34.28	1.64	0.13	0.42	51.69	7.4
	900 °C	1.09	2.51	0.66	0.99	35.57	2.04	0.15	0.55	52.12	8.4
	1100 °C	0.00	2.34	0.56	1.01	37.40	1.84	0.14	0.51	51.95	7.9
LP	105 °C	40.68	2.15	0.34	0.24	55.31	1.02	0.00	0.06	0.03	7.2
	600 °C	40.86	2.16	0.35	0.23	55.12	1.02	0.01	0.06	0.04	8.1
	900 °C	34.00	2.39	0.39	0.26	61.19	1.37	0.02	0.08	0.15	12.3
	1100 °C	0.00	4.49	0.49	0.37	91.04	2.55	0.03	0.05	0.77	12.3

^1^ Raw materials were burnt separately.

**Table 3:** Change in chemical composition at different ESA burning temperatures (%).

ESA	Ig. Loss	SiO_2_	Al_2_O_3_	Fe_2_O_3_	CaO	MgO	Na_2_O	K_2_O	SO_3_	pH
ESA 700 °C	12.11	13.23	7.79	2.50	33.08	0.90	0.24	0.73	25.87	11.7
ESA 800 °C	6.86	14.00	8.41	2.65	35.06	0.95	0.25	0.78	27.20	12.3
ESA 900 °C	2.04	13.65	8.07	2.86	38.78	1.02	0.23	0.74	28.63	12.4
ESA 1000 °C	0.00	16.86	9.86	3.06	39.82	1.25	0.30	0.81	24.17	11.8
ESA 1100 °C	0.00	16.98	9.30	3.28	44.42	1.24	0.33	0.91	19.48	11.2

**Table 4:** Change in chemical composition of OGE at different ESA burning temperatures (%).

OGE	Ig. Loss	SiO_2_	Al_2_O_3_	Fe_2_O_3_	CaO	MgO	Na_2_O	K_2_O	SO_3_
PBFSC ^1^	1.07	24.67	7.49	2.12	56.59	3.69	0.18	0.86	2.15
OGE 1 ^2^ (ESA 700 °C)	1.59	22.81	6.73	2.27	57.07	3.14	0.16	0.94	3.89
OGE 2 ^2^ (ESA 800 °C)	1.19	23.05	6.76	2.27	57.49	3.19	0.15	0.93	3.87
OGE 3 ^2^ (ESA 900 °C)	0.73	22.99	6.79	2.27	57.58	3.18	0.15	0.94	3.90
OGE 4 ^2^ (ESA 1000 °C)	0.00	23.26	7.01	2.32	57.63	3.17	0.17	0.96	3.92
OGE 5 ^2^ (ESA 1100 °C)	0.00	23.42	6.95	2.33	58.10	3.21	0.15	0.95	3.82

^1^ PBFSC (OPC + 70% + GBFS1 30%); ^2^ OGE (OPC + 70% + GBFS2 23% + ESA7%).

**Table 5:** Mix, compressive strength, and flow of mortar.

NO	Specimen	W/B (%)	Dosage (Mass Ratio)			Flow (mm)	Compressive Strength (MPa)				
			OPC	GBFS	ESA		1 day	3 day	7 day	14 day	28 day
1	OPC	50	100	0	0	174	10.7	27.4	39.7	48.9	52.8
2	PBFSC	50	70	30	0	193	6.0	20.1	35.0	47.8	58.3
3	OGE 1	50	70	23	7	176	9.8	25.6	40.0	48.7	56.3
4	OGE 2	50	70	23	7	176	11.2	28.1	41.1	51.3	58.4
5	OGE 3	50	70	23	7	175	9.3	26.1	38.0	48.9	57.5
6	OGE 4	50	70	23	7	179	9.3	23.9	36.5	45.6	52.1
7	OGE 5	50	70	23	7	180	9.8	22.3	34.0	45.2	58.3

**Table 6:** Table of cement paste mix.

NO	Specimen	W/B (%)	Dosage (Mass Ratio)			Curing Temperature (°C)
			OPC	GBFS	ESA	
1	OPC	50	100	0	0	20
2	PBFSC	50	70	30	0	20
3	OGE 1~5	50	70	23	7	20

**Table 7:** Items measured for each experiment.

Experiments	Measurement Items	Standard
Material (ESA)	XRD analysis	ASTM C457
Mortar	Slump flow	ASTM 1437-15
	Compressive strength	ASTM C109
Cement Paste	setting time by Vicat needle	ISO 9587
	Hydrates by XRD analysis	ASTM C457
	Hydrates by SEM analysis	-
	Free-lime	-

**Table 8:** Measurement of free-lime in OGE at different ESA burning temperatures.

NO	0.1 N HCl Consumption (mL)	Free Lime (%)
OPC	2.20	0.62
PBFSC	1.15	0.32
OGE 1	1.20	0.34
OGE 2	1.20	0.34
OGE 3	1.15	0.32
OGE 4	1.20	0.34
OGE 5	1.20	0.31
